# The Effect of Dental Implant Restoration on the Biomechanics of the Temporomandibular Joint in Patients with Posterior Tooth Loss: A Pilot Study

**DOI:** 10.3390/bioengineering12040419

**Published:** 2025-04-14

**Authors:** Yuanli Zhang, Chongzhi Yin, Fei Chen, Guizhi Zhang, Po Hao, Yongli Pu, Haidong Teng, Hong Huang, Zhan Liu

**Affiliations:** 1 Department of Medical Technology, Chongqing Three Gorges Medical College, Chongqing 404120, China; 20170037@cqtgmc.edu.cn (Y.Z.); lwzy007@yeah.net (C.Y.); 18883588053@163.com (G.Z.); hpo1979@126.com (P.H.); 20130004@cqtgmc.edu.cn (Y.P.); 2 Research Center of Oral Materials and Technology, Chongqing Three Gorges Medical College, Chongqing 404120, China; 3 Key Laboratory for Biomechanical Engineering of Sichuan Province, Sichuan University, Chengdu 610065, China; wchenfei2002@163.com (F.C.); tenghaidong@foxmail.com (H.T.); 4 Yibin Institute of Industrial Technology, Sichuan University Yibin Park, Yibin Lingang Economic and Technological Development Zone, Yibin 644600, China; 5 Department of Oral Implantology, Stomatological Hospital of Chongqing Medical University, Chongqing 401147, China; 500239@cqmu.edu.cn

**Keywords:** tooth loss, dental implants, temporomandibular joint (TMJ), temporomandibular disorders (TMDs), biomechanics, finite element analysis

## Abstract

Currently, controversy persists over whether dental implant restoration exacerbates or alleviates temporomandibular disorders (TMDs). This study aimed to analyze the impact of dental implant restoration on the biomechanics of the temporomandibular joint (TMJ) in patients with posterior tooth loss. Ten healthy volunteers (Control group) and twenty patients with posterior tooth loss (preoperative in the Pre group and postoperative in the Post group) were recruited. Three-dimensional maxillofacial models of the maxilla, mandible, dentition, and articular discs were reconstructed. The von Mises, contact, and tensile stresses of the TMJ were analyzed. Before implant restoration, the stresses of the TMJ in the Pre group were considerably higher than those in the Control group, especially on the missing tooth side. After restoration, the stresses in the Post group decreased significantly, with a near-symmetrical distribution. Additionally, before restoration, the patients with TMD had the highest stresses of the TMJ, followed by those without TMD, and the Control group had the lowest. After restoration, the stress magnitudes in the patients with or without TMD returned to the normal range. In summary, dental implant restoration can significantly improve the asymmetric stress distribution of the TMJs, substantially reduce excessive stress caused by tooth loss, and alleviate or eliminate the symptoms related to TMDs.

## 1. Introduction

Posterior tooth loss, a prevalent oral condition in clinical practice, can lead to significant functional impairments and esthetic issues, thereby diminishing the patients’ quality of life [1]. The incidence of posterior tooth loss tends to increase with age. According to certain research, among individuals aged 65 and above, around 30–50% experience posterior tooth loss [2]. The prevalent etiologies underlying posterior tooth loss encompass dental caries, periodontal disease, traumatic events, and genetic predispositions, along with several other factors [1]. Previous research has indicated that posterior tooth loss can cause alveolar bone resorption, which may potentially compromise the stability of the jawbone. Additionally, tooth loss can lead to the maxillary sinus invading the alveolar space and disturbing paranasal sinus function [3]. Multiple relevant studies have indicated that posterior tooth loss is among the primary causes of temporomandibular disorders (TMDs) [3,4]. TMDs encompass a set of musculoskeletal and/or neuromuscular dysfunctions that affect the temporomandibular joint (TMJ), masticatory muscles, and related tissues [5]. Related investigations have demonstrated that TMD is a common oral health concern, with an incidence rate ranging from 28% to 50% [6,7].

In comparison to restoration techniques such as fixed dental prostheses, removable partial dentures, and dental bridges, dental implant restoration has emerged as the preferred option for replacing missing posterior teeth. This is attributed to its distinct advantages, such as the absence of the need for grinding natural teeth, stable usage, robust performance, high efficiency, aesthetic appeal, and convenience [8,9,10]. However, the impact of dental implant restoration on occlusion remains unclear. Given the high prevalence of TMDs in the general population and the growing trend of oral rehabilitation using dental implants, the ongoing debate regarding whether dental implant therapy exacerbates or alleviates TMDs has become particularly timely.

Current studies have shown that TMD is related to the operation of prolonged mouth opening and the stability of implant prosthesis [11]. Several investigations have highlighted that one of the predominant causes of TMD in patients with posterior tooth loss undergoing implant restoration is the improper construction of maxilla–mandibular relationships. This encompassed incorrect centric relation, improper vertical dimension, and occlusal instability [5,12]. Additionally, some cases also reported the occurrence of TMJ-related pain after the implant restoration in edentulous patients [13].

Nevertheless, a contrasting perspective has been put forward by some scholars. They maintain that implant restoration can exert beneficial effects on augmenting the physiological functions of the TMJs [14,15,16,17]. Some studies have indicated that dental implant restoration has the capability to restore the normal occlusal vertical distance and to enhance the condyle position in the articular fossa, consequently diminishing the likelihood of TMD occurrence [14,15]. It has additionally been observed that implant repair can contribute to the rehabilitation of normal masticatory muscle function, the enhancement of the stability of the TMJ, and the exertion of a favorable influence on facilitating the normal physiological function of the TMJ [16,17]. Long-term clinical follow-up studies have gone a step further, uncovering that the incidence of TMD, such as joint pain, snapping, and restricted opening, is markedly reduced in patients following implant restoration [18,19,20].

The existing research regarding the impact of implant restoration on TMJ functions predominantly comprised clinical observations and imaging investigations [11,14]. The bilateral TMJs benefit from appropriate stress stimulation, which plays a crucial role in maintaining the normal dynamic remodeling process and physiological functions. The TMJ has been demonstrated to be a weight-bearing joint, and its physiological function is closely associated with its stress condition [21,22]. Either excessive or insufficient stress can precipitate the degenerative remodeling of the TMJ [23,24]. Consequently, biomechanical research is particularly important for understanding the impact of implant restoration on the function of the TMJs. This study is designed to analyze the disparities in the biomechanical parameters of the TMJ among patients with posterior tooth loss prior to and following implant restoration, and also in comparison with those of normal individuals. By doing so, it endeavors to explore the influence of implant restoration on the biomechanics within the TMJs, thereby providing a theoretical foundation for the treatment of implant restoration in patients with posterior tooth loss.

## 2. Materials and Methods

This study was carried out in strict accordance with the principles outlined in the Declaration of Helsinki. It received approval from the Ethics Committee of Chongqing Three Gorges Medical College (approval number: SYYZ-H-2302-0001). Prior to the commencement of the study, each subject was provided with comprehensive information about the study procedures, potential risks, and benefits. Subsequently, all of the subjects signed an informed consent form, which explicitly consented to their participation in the examinations and the use of all relevant data generated during the course of this study.

### 2.1. Patients and Imaging Data Acquisition

A total of 30 volunteers, all of whom were 18 years old or above, were recruited at the Affiliated Stomatology Hospital of Chongqing Medical University. An oral surgeon identified all of these subjects. Among them, 20 individuals (comprising 9 males and 11 females, with an average age of 48.05 ± 5.86 years) who had posterior tooth loss were designated as the preoperative group (abbreviated as the Pre group). The subjects in this group were those who had not yet undergone implant restoration. Those who had received implant restoration were categorized as the postoperative group (abbreviated as the Post group). Additionally, 10 healthy volunteers (including 5 males and 5 females, with an average age of 42.46 ± 4.89 years) with normal dentition were designated as the Control group.

For the study, the patients with posterior tooth loss had the following inclusion criteria: (1) the presence of maxillary or mandibular posterior tooth loss and no orthodontic treatment received prior to the experiment, (2) undergoing implant restoration with at least a 12-month follow-up, (3) no craniomaxillofacial or nervous system diseases (congenital or acquired), and (4) no history of mental illness or psychiatric drug use. They were excluded if they were under 18 years of age or diagnosed with jaw osteonecrosis. Regarding healthy volunteers with normal dentition, the inclusion criteria were as follows: (1) physical and mental health, no growth-related diseases; (2) facial and TMJ symmetry with no orthodontic or surgical history; (3) no TMD or other TMJ diseases. They were excluded if they were under 18 years of age or had a history of maxillofacial orthodontic or surgical treatment. In this study, all of the subjects were recruited and treated by a single, highly experienced dentist in strict accordance with the aforementioned inclusion and exclusion criteria. This approach guaranteed the consistency and reliability of both the research data collection and the treatment implementation.

All of the subjects were scanned using a CBCT machine (KaVo 3D eXam, KaVo Dental, Biberach an der Riss, Germany) following a standardized protocol to acquire head views. The scanning parameters were set as follows: a 16 × 11 cm field of view (FOV), 120 kVp, 5 mA, a voxel size of 0.4 mm, and a scanning speed of 360°/s for 4 s. Patients with posterior tooth loss underwent CBCT scans both before and six months after implant restoration. The cross-sectional images had a resolution of 400 pixels and a pixel size of 0.4 mm. Each CBCT scan generated 290 to 340 images with a slice thickness of 0.4 mm. Subsequently, the CBCT data were converted into the Digital Imaging and Communications in Medicine (DICOM) format.

**Figure 1:**
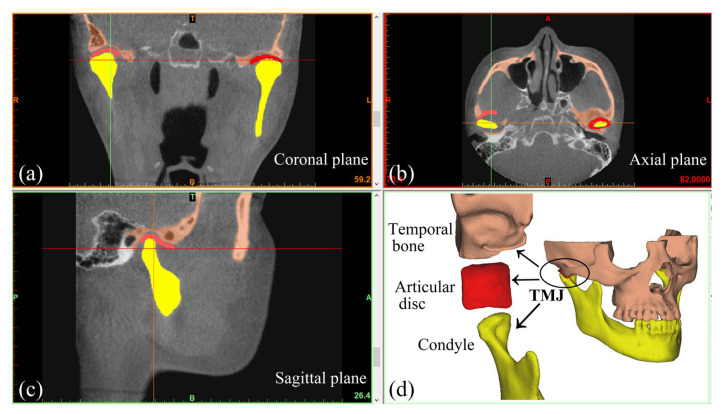
Reconstruction of maxillofacial 3D model. (**a**) The model reconstructed in the coronal plane. (**b**) The model reconstructed in the transverse plane. (**c**) The model reconstructed in the sagittal plane. (**d**) The maxillofacial 3D model (including the TMJ) calculated and generated based on the image information in three dimensions. Yellow represents the mandible, red represents the articular disc, and brown represents the maxilla. “T” denotes the top, “B” the bottom, “R” the right side, “L” the left side, “A” the anterior, and “P” the posterior.

**Figure 2:**
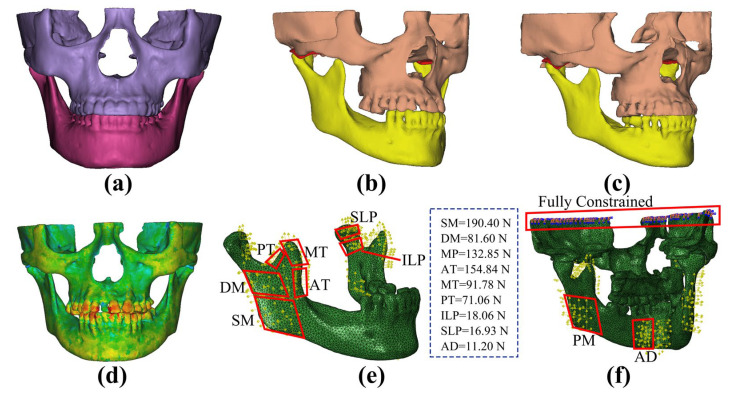
Finite element models and parameter settings for healthy volunteers and patients with posterior tooth loss. (**a**) Finite element model of a healthy volunteer (Control group). (**b**) Finite element model of the patient with posterior tooth loss before restoration (Pre group). (**c**) Finite element model of the patient with posterior tooth loss after implant restoration (Post group). (**d**) The material parameters, as follows: the density and elastic modulus of the bones and teeth defined according to the Hounsfield units (red and blue represent the maximum and minimum density and elastic modulus, respectively). (**e,f**) Muscle forces and boundary constraints in the finite element models (SM—superficial masseter, DM—deep masseter, MP—medial pterygoid, AT—anterior temporalis, MT—middle temporalis, PT—posterior temporalis, ILP—inferior head of lateral pterygoid, SLP—superior head of lateral pterygoid, and AD—anterior digastric muscle. The red boxes located on the mandible represent the attachment areas for muscular forces, while the red box positioned at the top of the maxilla indicates the fixed constraint region.).

Based on grayscale values, three-dimensional (3D) models of the subjects’ maxilla, mandible, and teeth were reconstructed using Mimics (Mimics Innovation Suite, Materialise, Leuven, Belgium) (Figure 1). The discs were created according to MRI images (SIGNA HDe, GE, Milwaukee, WI, USA) and anatomical features. Thus, 3D models of various subjects were successfully obtained (Figure 1 and Figure 2).

### 2.2. Finite Element Model

In this study, ABAQUS software (ABAQUS 6.13, Dassault SIMULIA, Providence, RI, USA) was adopted to conduct the finite element analysis and calculations. Referring to the empirical formula between the material properties of bone and the grayscale value (Equations (1) and (2)), the density and elastic modulus were assigned to the maxilla and mandible. This was performed to simulate the heterogeneous material properties of bone tissue [25,26] (Figure 2d). Poisson’s ratio of the maxilla and mandible was assigned as 0.3 [22,27]. The disc was considered as a Mooney–Rivlin hyperelastic material, with the parameters *C*_1_ = 9 × 10^5^ Pa and *C*_2_ = 9 × 10^2^ Pa. Previous studies have demonstrated that this material is well suited for the finite element analysis of the TMJ [28,29,30,31]. The strain–energy function of this material was defined as in Equation (3). Furthermore, the articular cartilages with a thickness of 0.2 mm were established on the surfaces of the condyle and the articular eminence, with an elastic modulus of 0.79 MPa and Poisson’s ratio of 0.49 [22].

*Density* = −13.4 + 1017 × *GV* (1)

*E* = −388.8 + 5925 × *Density* (2)

*W*(*I*_1_, *I*_2_) = *C*_1_(*I*_1_ − 3) + *C*_2_(*I*_2_ − 3) (3)

where *GV* is the grayscale value, *E* is the elastic modulus, *C*_1_ and *C*_2_ are material constants, and *I*_1_ and *I*_2_ are the first and second invariants of the left Cauchy–Green deformation tensor B.

To enhance both the accuracy and efficiency, the modified quadratic tetrahedron element (C3D10M) was employed for the TMJ regions, while the 4-node linear tetrahedron element (C3D4) was utilized for the remaining regions of the models. The model consisted of approximately 190,000 elements and 80,000 nodes, respectively (Figure 2f).

A surface-to-surface contact approach was adopted to simulate the interaction between contact surfaces. Moreover, a hard contact condition was set with a frictional coefficient of 0.001 [22]. Nonlinear cable elements were used to model the following four disc attachments: the anterior temporal attachment, the anterior mandibular attachment, and the upper and lower bands of the bilaminar zone [32]. The numbers of these elements for each attachment were 4, 4, 6, and 5, respectively.

### 2.3. Load Conditions

The static occlusion was analyzed in this study. Identical loading conditions were imposed on all of the models. The temporomandibular, sphenomandibular, and stylomandibular ligaments were modeled as nonlinear axial connectors [33]. Nine primary muscle forces were taken into account, with the magnitudes and directions of the muscle forces during centric occlusion determined based on prior research (Table 1) [34]. The top surface of the maxilla was fully constrained (Figure 2f).

### 2.4. Statistical Analysis

Statistical analysis was carried out using SPSS 27.0 (SPSS Statistics 27.0, IBM, Armonk, NY, USA). To compare the differences in biomechanical parameters between the Pre and Control groups, as well as between the Post and Control groups, the independent sample *t*-test was employed. However, when the data failed to exhibit a normal distribution pattern, the Mann–Whitney *U* test was employed.

For comparing the differences in the biomechanical parameters between the Pre and Post groups, the paired-samples *t*-test was used. Additionally, this pair of tests was also applied to compare the differences in biomechanical parameters between the healthy side and the missing tooth side/implant restoration side of the Pre and Post groups. Similarly, when the data did not follow a normal distribution, the Mann–Whitney *U* test was employed.

Statistical significance was achieved when *p* < 0.05.

## 3. Results

The temporomandibular joint functions as a weight-bearing joint, with its load-bearing characteristics varying under different oral conditions, including healthy dentition, missing posterior teeth, and implant restoration following tooth loss. The von Mises stress was selected to represent the overall stress distribution within the TMJ. Tensile stress was utilized to assess the tensile damage to the bone and soft tissues, while contact stress was employed to analyze the compression between the articular disc and condyle, as well as between the articular disc and temporal bone.

**Figure 3:**
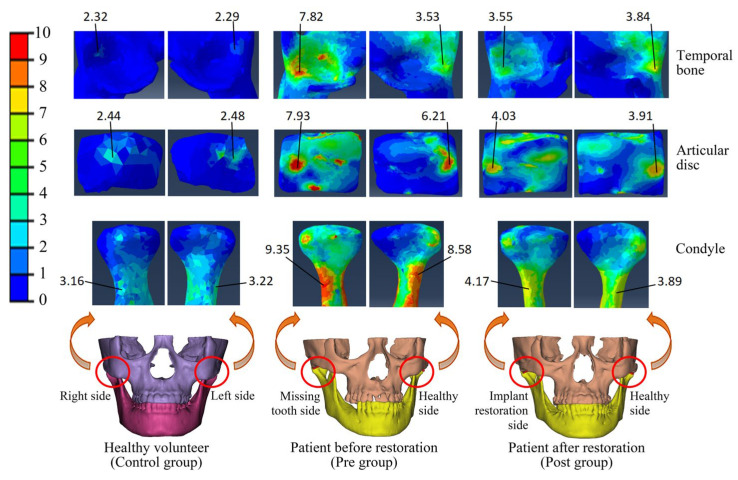
The von Mises stress distribution in the temporal bone, articular disc, and condyle of healthy volunteer and patients before and after implant restoration under the centric occlusion (unit: MPa). Red indicates the high-stress regions and blue indicates the low-stress regions.

The von Mises stress serves as an indicator of the overall stress state within the TMJ. This study revealed that the patients with posterior tooth loss (Pre group) commonly exhibited stress concentrations in the articular disc, condyle, and temporal bone, with an asymmetrical stress distribution between the left and right TMJs (Figure 3). However, after implant restoration (Post group), the stresses on the patients’ TMJs became relatively uniform and basically returned to the normal level, and the stress distributions on both sides were symmetrical (Figure 3).

**Figure 4:**
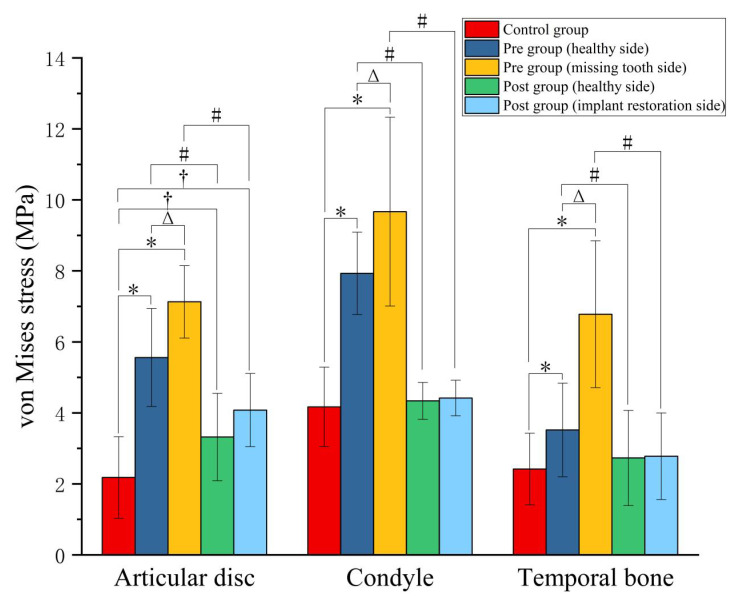
The average von Mises stress of the articular disc, condyle, and temporal bone in the Control, Pre, and Post groups (unit: MPa). * indicates a statistically significant difference between the Control group and Pre group (healthy side/missing tooth side) (*p* < 0.05); Δ indicates a statistically significant difference between the healthy side and missing tooth side of the Pre group (*p* < 0.05); † indicates a statistically significant difference between the Control group and Post group (healthy side/implant restoration side) (*p* < 0.05); # indicates a statistically significant difference between the Pre group and Post group (compared with the same side) (*p* < 0.05).

Through a comparison of the von Mises stress differences among patients with posterior tooth loss before restoration (Pre group), after restoration (Post group), and healthy individuals (Control group), it was revealed that the stress magnitudes in the articular disc, condyle, and temporal bone on the missing tooth side of the Pre group were significantly greater than those on the healthy side (*p* < 0.05). Specifically, the differences between the two sides were 28.24%, 21.94%, and 92.61% for the articular disc, condyle, and temporal bone, respectively (Figure 4). After implant restoration, the von Mises stress magnitudes of each TMJ structure in the Post group were comparable between the implant restoration side and healthy side, with no statistically significant differences. Furthermore, the stress magnitudes in the articular disc, condyle, and temporal bone of the Pre group were also significantly greater than those of the Control group (*p* < 0.05), being 3.27 times, 2.32 times, and 2.80 times higher, respectively. Subsequent to implant restoration, the stress magnitudes of the condyle and temporal bone in the Post group were reduced to the normal range. Although the von Mises stress magnitudes of the articular disc decreased from 3.27 times to 1.87 times the normal value, it still remained significantly higher compared to the Control group (Figure 4).

**Figure 5:**
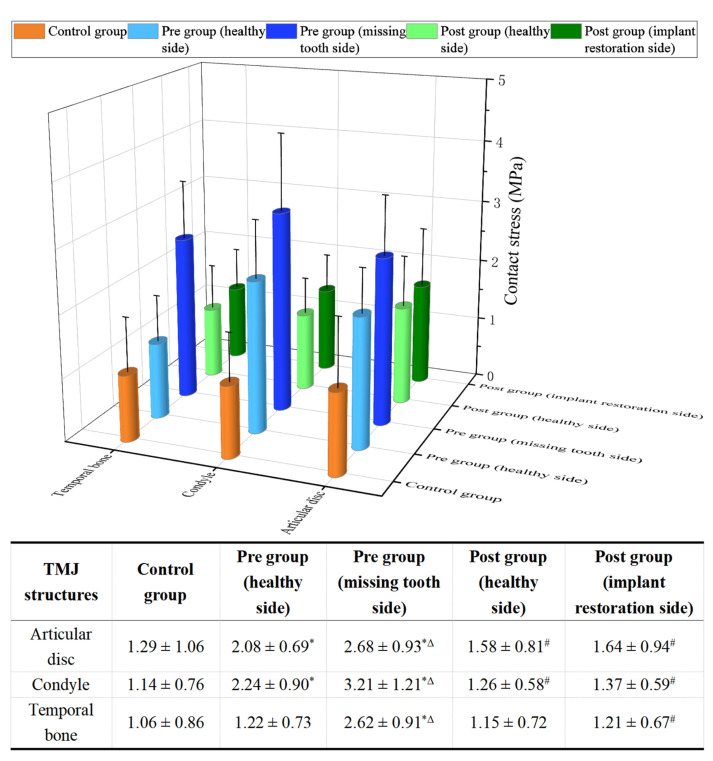
The average contact stress of the articular disc, condyle, and temporal bone in the Control, Pre, and Post groups (unit: MPa). * statistically significant difference between the Control group and Pre group (healthy side/missing tooth side) (*p* < 0.05); Δ statistically significant difference between the healthy side and missing tooth side of the Pre group (*p* < 0.05); # statistically significant difference between the Pre group and Post group (compared with the same side) (*p* < 0.05).

This study revealed that, prior to implant restoration, the patients with posterior tooth loss (Pre group) exhibited significantly higher contact stress in the articular disc, condyle, and temporal bone compared to the Control group (*p* < 0.05), particularly on the side of the missing tooth (Figure 5). Following implant restoration, there were marked reductions in the contact stresses of the articular disc, condyle, and temporal bone, decreasing by 38.81%, 57.32%, and 53.82%, respectively, compared to pre-restoration levels (Figure 5). Additionally, after the implant restoration, the contact stress on both TMJs (Post group) became nearly equal and exhibited no significant statistical difference from the Control group (Figure 5).

**Figure 6:**
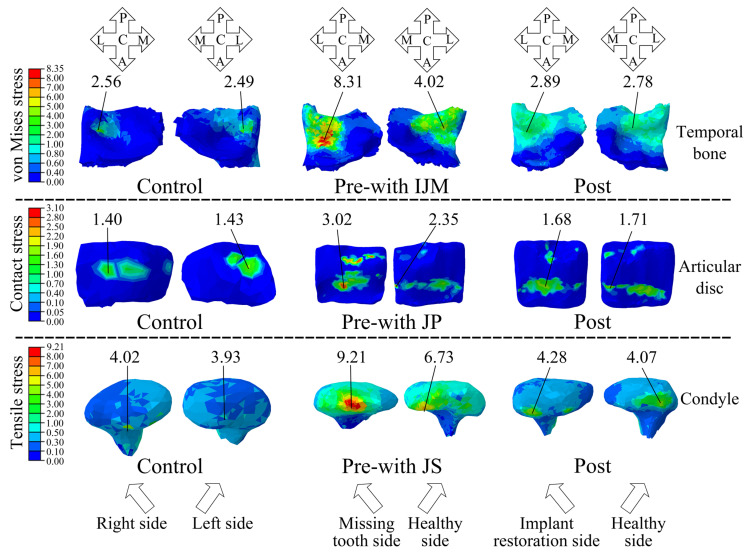
The von Mises stress, contact stress, and tensile stress distributions of the TMJ (unit: MPa). Control represents the healthy volunteer; Pre-with IJM represents the patients with inconsistent joint mobility before implant restoration, Pre-with JP represents the patients with joint pain before implant restoration, and Pre-with JS represents the patients with joint sounds; Post represents the corresponding patients after dental implant restoration; A represents anterior; P represents posterior; C represents central; L represents lateral; M represents medial. Red indicates the high-stress regions and blue indicates the low-stress regions.

Prior to implant restoration, a majority of patients with posterior tooth loss exhibited TMD symptoms. Specifically, 15 cases presented with inconsistent joint mobility, 12 cases reported joint pain, and 13 cases experienced joint sounds. Significant differences in the magnitudes and distributions of stress were observed between these patients and the Control group. This study revealed that, for the patients with inconsistent joint mobility (the Pre-with IJM), the von Mises stress concentration in the temporal bone was predominantly located in the glenoid fossa, with stress magnitudes notably exceeding normal levels (as seen in the Control group) (Figure 6). After implant restoration, the TMD symptoms in the patients were greatly improved, with only three patients still having slight symptoms of joint pain. Meanwhile, the magnitudes of the von Mises stress in the temporal bone of these patients were largely normalized, and their stress distribution was aligned with that of the Control group, with a concentration on the posterior slope of the articular tubercle (Figure 6).

For the patients experiencing joint pain prior to implant restoration (the Pre-with JP), high-contact-stress regions on the articular disc were primarily found in the anterior and posterior bands. Following implant restoration, the contact stress on the articular disc was significantly reduced to normal ranges (as seen in the Control group); however, the elevated stress regions remained concentrated in the anterior band of the articular disc. This stress distribution contrasts with that observed in the Control group, where the high-stress regions were primarily located in the intermediate zone of the articular disc (Figure 6).

This study revealed that the patients with joint sounds (Pre-with JS) exhibited significantly elevated condylar tensile stress levels compared to normal values (as seen in the Control group), predominantly localized in the transverse ridge of the condyle. Following implant restoration, the condylar tensile stress in these patients was markedly reduced and returned to nearly normal levels. The stress distribution pattern also aligned with that of the Control group, with a concentration on the anterior slope of the condyle (Figure 6).

For the patients with posterior tooth loss without TMD symptoms, the stress magnitudes in the TMJ structures were lower than those with TMD symptoms prior to implant restoration. However, these pretherapeutic magnitudes were still significantly higher than those of the Control group. Additionally, it also showed that the stress magnitude on the missing tooth side was greater (Figure 6 and Table 2). Following implant restoration, the stress levels in the TMJ structures were significantly reduced compared to pre-restoration levels, without a statistically significant difference observed relative to the Control group (Table 2).

## 4. Discussion

The aim of this study was to investigate the impact of dental implant restoration on the biomechanical parameters (von Mises stress, contact stress, and tensile stress) of the TMJ in the patients with posterior tooth loss. Specifically, this research explored the changes in these biomechanical parameters in the patients with and without TMD symptoms before and after implant restoration. The findings are intended to provide a theoretical foundation for optimizing clinical applications of implant restoration in patients with posterior tooth loss.

In our previous studies [33,35], the accuracy of the simulation was verified through the experiments using five 3D-printed models. The finite element and experimental models shared the same geometries, material properties, load conditions, and boundary conditions. The maximum differences between the measured strains (from the 3D-printed models) and the calculated strains (from the finite element models) were less than 5% [35]. Furthermore, in this study, the maximum contact stress of the articular discs of healthy volunteers was found to be 1.29 MPa, consistent with the similar research findings of Zhu et al. [32]. Additionally, this study found that the maximum von Mises stress of the condyles of healthy volunteers was 4.17 MPa, and related similar studies have shown that the maximum von Mises stress of the condyles of healthy volunteers was 4.21 MPa [36]. Therefore, the simulation method was reasonable and the results were accurate in this study.

This research uncovered that the loss of posterior teeth would trigger a significant elevation in the stress of the TMJ, especially on the side with tooth loss. Specifically, the maximum von Mises stresses of the temporal bone, articular disc, and condyle on the missing tooth side were 3.27, 2.32, and 2.80 times greater than those recorded in the normal Control group, respectively (Figure 3 and Figure 4). According to Wolff’s law [24], excessive stress prompts an enhancement in the activity of osteoblasts and stimulates bone proliferation. This, in turn, leads to the expansion of the bearing area and facilitates the establishment of a new physiological equilibrium. The condyle and the temporal bone are predominantly composed of hydroxyapatite and collagen fibers [37]. In contrast, the articular disc mainly consists of fibrous connective tissue [38]. Under normal physiological loading conditions, both the bone structures and the articular disc exhibit excellent wear resistance and a high fatigue strength [37,38]. Nevertheless, when the von Mises stress surpasses their respective fatigue-related stress thresholds, plastic deformation or fatigue failure is likely to occur [37,38]. This study revealed that regions with higher von Mises stress coincide with areas of wear and fatigue damage. These findings offer valuable insights into the biomechanical mechanisms underpinning the pathologies of the TMJ. By comparing the von Mises stresses of the temporal bones in the patients exhibiting inconsistent joint mobility symptoms and those without TMD symptoms, it was revealed that the patients with TMD symptoms displayed greater stress deviations on both TMJs. These findings further corroborated the fact that the inconsistent stress distribution across the TMJs would result in the disparate remodeling of the joint surface. Specifically, the side subjected to greater stress exhibits a larger bearing area after joint remodeling, which results in structural asymmetry and inconsistent joint mobility between both TMJs. This conclusion is supported by clinical follow-up records, which indicated that 15 out of 20 patients exhibited the symptoms associated with inconsistent joint mobility prior to implant restoration.

Following implant restoration, a remarkable reduction was observed in the von Mises stresses of all TMJ structures, with magnitudes largely returning to the normal range. Concurrently, the stress distribution on both TMJs reverted from an asymmetric to a symmetric state. Clinical follow-up records also verified that, after the implant procedure, the symptoms of inconsistent joint mobility on both TMJs in the patients had vanished completely. These results indicated that restoring normal stress levels in the TMJ can re-establish physiological equilibrium, which suggests a close relationship between the stress distribution and TMJ function.

Furthermore, by combining the observations of TMJ stress changes before and after implant restoration with clinical manifestations of TMD symptoms, we found that, when the TMJ stress distributions were asymmetrical and the stress magnitudes were significantly higher than normal (as seen in the Control group), progressive asymmetric remodeling of the TMJ bone tissue occurred. The joint surface area with higher stress increased after reconstruction, leading to bilateral TMJ asymmetry and clinical signs of inconsistent joint mobility. After implant restoration, the stress distribution of the bilateral TMJs in the patients became essentially symmetric, and the stress levels returned to the normal range. As a result, the TMJ remodeling resumed its normal physiological balance, and the bilateral TMJs became predominantly symmetric after remodeling. Consequently, TMD symptoms such as inconsistent joint mobility, which were present prior to restoration, disappeared. The outcomes of this study demonstrated that implant restoration could effectively diminish the stress level of the TMJs in patients with posterior tooth loss and optimize the symmetric stress distributions of the bilateral TMJs. This enabled the TMJ remodeling to return to its normal physiological equilibrium and thereby achieved the clinical objective of eliminating inconsistent joint motion.

As is commonly known, contact stress indicates the degree of extrusion between two objects. This study found that posterior tooth loss caused the contact stresses of the bilateral TMJs to be significantly higher than those of the Control group, particularly on the side with the missing tooth. There, the contact stresses of the articular disc, condyle, and temporal bone were 2.08, 2.82, and 2.47 times those of the Control group, respectively. These findings demonstrate that an elevation in the contact stress on both the bone tissue and articular disc within the TMJ results in a decline in their wear resistance and fatigue strength [39]. Such alterations are intricately linked to the symptoms manifested in the TMJ. For instance, the reduction in the fatigue strength of the articular disc is correlated with intensified joint pain and the emergence of abnormal joint noises, a phenomenon corroborated by the follow-up records of this study. After implant restoration, the contact stresses on both TMJs were significantly reduced, with those of the articular disc, condyle, and temporal bone being 1.27, 1.20, and 1.14 times those of the Control group, which are slightly above the normal range. The clinical follow-up of the 20 patients with posterior tooth loss showed that 12 patients exhibited joint pain before restoration. Following implant restoration, only three patients still had slight joint pain, while the rest had no such symptoms. These results suggested a close correlation between biomechanical parameters and clinical symptoms. Posterior tooth loss aggravated the extrusion between the condyle and the articular disc, and between the temporal bone and the articular disc, and thus increased the pressure within the TMJ, leading to joint pain. After restoration, the contact levels among the TMJ structures were greatly relieved, basically returning to a normal physiological state, and thus the preoperative joint pain symptoms were alleviated or gone.

Does the presence or absence of TMD symptoms among the patients with posterior tooth loss exert an impact on the stresses within the TMJs? This study has clearly demonstrated that such an impact undoubtedly exists. This study found that, prior to implant restoration, the magnitudes of the von Mises stress, contact stress, and tensile stress were the highest in the patients with posterior tooth loss accompanied by TMD symptoms (such as inconsistent joint mobility, joint pain, and joint sounds), followed by those patients with posterior tooth loss but without TMD symptoms, and were the lowest in the Control group (Figure 6 and Table 2). Furthermore, in the patients with posterior tooth loss, regardless of whether they had TMD or not, the stresses on the side of the missing teeth were relatively higher. These results suggested that both posterior tooth loss and TMD can lead to a significant increase in the stress on the TMJs.

After implant restoration, the stresses in the patients with posterior tooth loss, whether they had TMD or not, decreased significantly and basically reverted to the normal range (Figure 6 and Table 2). Additionally, the clinical follow-up records of this study revealed that, among the 20 patients with posterior tooth loss recruited herein, the symptom of inconsistent joint mobility completely vanished in the 15 patients of the “Pre-with IJM” group. Similarly, the symptom of joint sounds completely disappeared in the 13 patients of the “Pre-with JS” group. This might be associated with the fact that, after implant restoration, the stresses of the patients in the “Pre-with IJM” group and the “Pre-with JS” group dropped to 1.08 times and 1.01 times those of the Control group, respectively, from 3.25 times and 2.29 times those of the Control group. The deviations of the stress magnitudes of the above two groups of patients from the normal range (the magnitudes in the Control group) were all within 10% after implant restoration. Moreover, among the 12 patients with joint pain in the “Pre-with JP” group, the symptom completely disappeared in 9 patients after implant restoration, while 3 patients still had a slight degree of joint pain. This might be related to the fact that the stress in the patients of the “Pre-with JP” group decreased from 2.16 times to 1.22 times those of the Control group. The stress of such patients still deviated from the normal level by 22%, so, clinically, there were still three patients who still had slight joint pain after implant restoration. Relevant studies have also pointed out that excessive stress on the TMJ would lead to TMD symptoms [40,41].

Limitations and Prospects: In the present study, the exclusive inclusion of participants from a single medical institution potentially restricts the generalizability of the research outcomes. With a relatively small sample size of only 20 patients, there is a likelihood of reducing the statistical power to a certain degree, thereby impeding the extrapolation of the results. The follow-up duration was merely 12 months. While this allowed for a reasonable assessment of the short-term impacts of implant restoration on the TMJ biomechanics and TMD symptoms, it was inadequate for evaluating the lasting effects and long-term stability of the results. Furthermore, the current investigation was centered on static occlusal conditions, leading to an incomplete understanding of the TMJ’s dynamic mechanisms. For future research endeavors, the implementation of multi-center studies, an increase in the sample size, and an extension of the follow-up period to 3–5 years are recommended. These steps would enhance the reliability, generalizability, and long-term stability of the research findings. Additionally, future studies could incorporate dynamic loading conditions, such as mandibular opening and closing movements, to foster a more profound and comprehensive comprehension of the dynamic mechanisms of the TMJ.

## 5. Conclusions

This study investigated the alterations in TMD symptoms among patients with posterior tooth loss before and after implant restoration by analyzing the biomechanical parameter differences in the TMJ and their deviations from normative values. The findings demonstrated a strong correlation between the TMJ stress magnitude and TMD symptoms, where excessive mechanical stress may precipitate TMD symptoms such as joint pain. Furthermore, the research highlights that implant restoration could effectively restore the TMJ stress distribution to physiological levels in patients with posterior tooth loss, thereby alleviating or eliminating TMD symptoms.

In conclusion, dental implant restoration can significantly improve the asymmetric stress distributions of the TMJs in patients with posterior tooth loss and remarkably reduce the excessive stress on the TMJ caused by tooth loss, thus enabling it to basically return to the normal range. Moreover, implant restoration can also alleviate or eliminate the TMD symptoms among patients with posterior tooth loss, including joint pain, inconsistent joint mobility, and joint sounds. This study establishes a theoretical foundation for implant restoration in patients with posterior tooth loss and the prevention/management of TMD.

## Figures and Tables

**Table 1:** The magnitudes and directions of the muscular forces, along with the efficiency (contribution) of these forces under centric occlusion.

	Maximum Muscle Force (N)	Cos-x		Cos-y		Cos-z		Efficiency (Centric Occlusion)
		L	R	L	R	L	R	
SM	190.40	0.21	−0.21	−0.42	−0.42	0.88	0.88	1.00
DM	81.60	0.55	−0.55	0.36	0.36	0.76	0.76	1.00
MP	174.80	−0.49	0.49	−0.37	−0.37	0.79	0.79	0.76
AT	158.00	0.15	−0.15	−0.04	−0.04	0.99	0.99	0.98
MT	95.60	0.22	−0.22	0.50	0.50	0.84	0.84	0.96
PT	75.60	0.21	−0.21	0.86	0.86	0.47	0.47	0.94
ILP	66.90	−0.63	0.63	−0.76	−0.76	−0.17	−0.17	0.27
SLP	28.70	−0.76	0.76	−0.65	−0.65	0.07	0.07	0.59
AD	40.00	0.24	−0.24	0.94	0.94	−0.24	−0.24	0.28

Note: SM indicates superficial masseter; DM indicates deep masseter; MP indicates medial pterygoid; AT indicates anterior temporalis; MT indicates middle temporalis; PT indicates posterior temporalis; ILP indicates inferior head of lateral pterygoid; SLP indicates superior head of lateral pterygoid; AD indicates anterior digastric muscle; Cos-x, Cos-y, and Cos-z, respectively, represent the cosine values of the angles between the direction of the muscular force in the three-dimensional space and the positive directions of the *X*-axis, *Y*-axis, and *Z*-axis; L and R represent the left side and the right side of the maxillofacial model, respectively.

**Table 2:** The average stress (MPa) in the high-stress regions of healthy volunteers (Control group) and the patients without TMD before and after implant restoration.

	Control Group	Pre-Without TMD Group (Missing Tooth Side)	Pre-Without TMD Group (Healthy Side)	Post Group (Implant Restoration Side)	Post Group (Healthy Side)
von Mises stress of temporal bone	2.42 ± 1.01	5.93 ± 1.15 (*p* = 0.000) *	3.21 ± 0.86 (*p* = 0.001) *, (*p* = 0.000) ^Δ^	2.73 ± 1.07 (*p* = 0.000) ^#^	2.69 ± 1.12 (*p* = 0.001) ^#^
Contact stress of articular disc	1.29 ± 1.06	2.29 ± 0.54 (*p* = 0.001) *	1.72 ± 0.33 (*p* = 0.013) *, (*p* = 0.009) ^Δ^	1.59 ± 0.75 (*p* = 0.002) ^#^	1.51 ± 0.68 (*p* = 0.198)
Tensile stress of condyle	3.90 ± 0.82	7.92 ± 1.57 (*p* = 0.000) *	5.81 ± 0.42 (*p* = 0.000) *, (*p* = 0.000) ^Δ^	4.15 ± 0.42 (*p* = 0.000) ^#^	4.01 ± 0.44 (*p* = 0.000) ^#^

Note: * statistically significant difference between the Control group and Pre-without TMD group (missing tooth side/healthy side) (*p* < 0.05); Δ statistically significant difference between the healthy side and missing tooth side of the Pre-without TMD groups (*p* < 0.05); # statistically significant difference between the Pre-without TMD group and Post group (compared with the same side) (*p* < 0.05). No statistically significant differences were observed in the following comparisons: (1) between the Post group (implant restoration side) and the Control group; (2) between the Post group (healthy side) and the Control group; (3) between the Post group (implant restoration side) and the Post group (healthy side).

## Data Availability

The original contributions presented in this study are included in the article. Further inquiries can be directed to the corresponding author.

## Abbreviations

The following abbreviations are used in this manuscript:

TMJ	Temporomandibular joint
TMDs	Temporomandibular disorders
CBCT	Cone beam computed tomography
FOV	Field of view
DICOM	Digital Imaging and Communications in Medicine
3D	Three-dimensional
MRI	Magnetic resonance imaging
GV	Grayscale value
C3D10M	Modified quadratic tetrahedron element
C3D4	Four-node linear tetrahedron element
SM	Superficial masseter
DM	Deep masseter
MP	Medial pterygoid
AT	Anterior temporalis
MT	Middle temporalis
PT	Posterior temporalis
ILP	Inferior head of lateral pterygoid
SLP	Superior head of lateral pterygoid
AD	Anterior digastric muscle
